# Suppressing Klein tunneling in graphene using a one-dimensional array of localized scatterers

**DOI:** 10.1038/srep08435

**Published:** 2015-02-13

**Authors:** Jamie D. Walls, Daniel Hadad

**Affiliations:** 1 Department of Chemistry, University of Miami, Coral Gables, Florida 33124, USA

## Abstract

Graphene's unique physical and chemical properties make it an attractive platform for use in micro- and nanoelectronic devices. However, electrostatically controlling the flow of electrons in graphene can be challenging as a result of Klein tunneling, where electrons normally incident to a one-dimensional potential barrier of height *V* are perfectly transmitted even as *V* → ∞. In this study, theoretical and numerical calculations predict that the transmission probability for an electron wave normally incident to a one-dimensional array of localized scatterers can be significantly less than unity when the electron wavelength is smaller than the spacing between scatterers. In effect, placing periodic openings throughout a potential barrier can, somewhat counterintuitively, decrease transmission in graphene. Our results suggest that electrostatic potentials with spatial variations on the order of the electron wavelength can suppress Klein tunneling and could find applications in developing graphene electronic devices.

Since graphene's initial discovery^1^, much research has been undertaken into studying graphene's unique physical and chemical properties^2^,^3^, which are a consequence of its two-dimensional structure. Graphene consists of carbon atoms arranged in a honeycomb lattice made up of two trigonal sublattices that each contribute a carbon atom to the unit cell, thereby imparting a pseudospin character to the electrons in graphene. From tight-binding calculations^4^, the quasiparticle spectrum of graphene is linearly proportional to the magnitude of the wave vector, 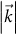, when expanded about two distinct wave vectors, 
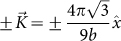
 with *b* = 1.42 *Å* being the C-C bond length. The wave vectors 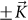 are referred to as Dirac points due to the similarity of the electronic spectrum in graphene to that of a massless two-dimensional Dirac fermion^5^. A consequence of the linear dispersion and the pseudospin nature of electron waves in graphene is Klein tunneling^6^, where massless Dirac fermions normally incident to a potential step barrier are not reflected even when the potential barrier height becomes infinite [Figure 1(A)]. Klein tunneling makes it difficult to stop the flow or transmission of electrons electrostatically, which poses a significant challenge for incorporating graphene into new electronic devices.

One proposed method for controlling and modifying the electronic properties in graphene has been to use superlattice potentials, 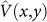. In this case, graphene's effective Hamiltonian, when expanded about the 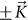 Dirac points, is given by^7^:
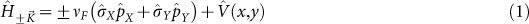
where 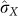 and 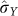 are pauli spin matrices, 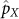 and 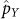 are momentum operators, and 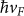 = 1.0558 × 10^−28^ J-m. In writing Eq. (1), the spatial variations of 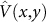 are assumed to be on length scales much greater than the C-C bond length. Such superlattice potentials can, in principle, be patterned on graphene using either localized chemical modifications^8^,^9^ or by locally manipulating the voltages of metallic islands or electrodes^10^,^11^ placed on top of graphene. Previous theoretical work^12^,^13^,^14^,^15^,^16^,^17^ has mainly focused on using periodic potentials along a single dimension, e.g., a periodic array of square potential barriers like the one shown in Fig. 1(A). For such a Kronig-Penney potential, there is no suppression of Klein tunneling for electrons at normal incidence. Other types of superlattice potentials, such as the muffin-tin superlattice potential^12^,^18^, which consists of a two-dimensional array of cylindrically symmetric step potentials, have been theoretically shown to generate electron localization and significantly alter graphene's dispersion relationship although the transport properties through such superlattice potentials have not been examined.

For single or multiple square potential barriers, Klein tunneling is still not suppressed since normally incident waves are only allowed to undergo direct backscattering (specular reflection) from such potentials, which is prohibited due to time-reversal symmetry in graphene. Therefore, to suppress Klein tunneling, electrostatic potentials are required that generate non-specular or diffuse reflection, which is not prohibited by time-reversal symmetry. One such potential that can exhibit diffuse reflection is a one-dimensional periodic array of localized scattering potentials as shown in Figure 1(B). Such potentials appear often in optics and in atomic/matter wave diffraction experiments and can be patterned on graphene^9^. As shown in this work, when the electron's wavelength, *λ*, becomes comparable to the spacing between scatterers, *d*, non-specular reflection can lead to a dramatic reduction in the transmission through such potentials even at normal incidence. As a result, Klein tunneling in graphene can be suppressed when using a periodic array of localized scatterers.

## Results

In the absence of an electrostatic potential 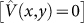, the positive energy solutions to Eq. (1) with energy 
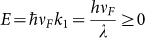
 and normalized to unit flux along the 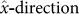 are the Dirac plane wave spinors 
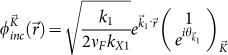
 and 
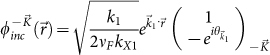
 with wave vector 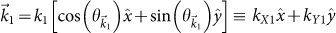, wavelength 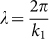, and 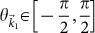. Consider a Dirac plane wave spinor 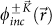 incident to an array of localized cylindrically symmetric scattering potentials as depicted in Fig. 2(A). Each scatterer is modeled by a simple step potential with an effective radius of *r_s_* so that the potential for the *n^th^* scatterer is given by 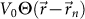 where 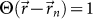 if 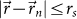 and 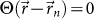 for 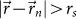. To consider only intravalley scattering 
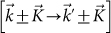
 and to neglect intervalley scattering 
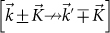
, *r_s_* must be greater than the C-C bond length in graphene, i.e., 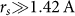.

As derived in Supporting Information, the transmitted wave function 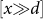, 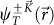, can be written as a sum of Dirac plane wave spinors with wave vectors along the Bragg directions, 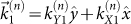 for integer *n* where 
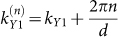
 and 
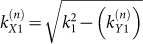
 for 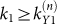. In this case, the transmitted wave function through the one-dimensional array of localized scatterers can be written as a sum over Dirac plane wave spinors propagating along the Bragg directions for 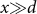 as:
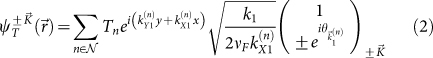
where *T_n_* is the transmission coefficient for the *n^th^* Bragg direction or open scattering channel. The sum in Eq. (2) is over all open scattering channels, 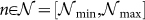 where 
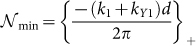
 and 
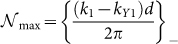
, where {*z*}_+_ corresponds to the smallest integer greater than *z*, and {*z*}_−_ corresponds to the largest integer less than *z*.

Likewise, the reflected wave function 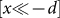 is given by:
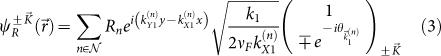
where *R_n_* is the reflection coefficient for the *n^th^* open scattering channel in 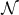. Due to the unitarity condition, 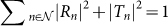. Expressions for *R_n_* and *T_n_* are given in Supporting Information.

**Figure 1:**
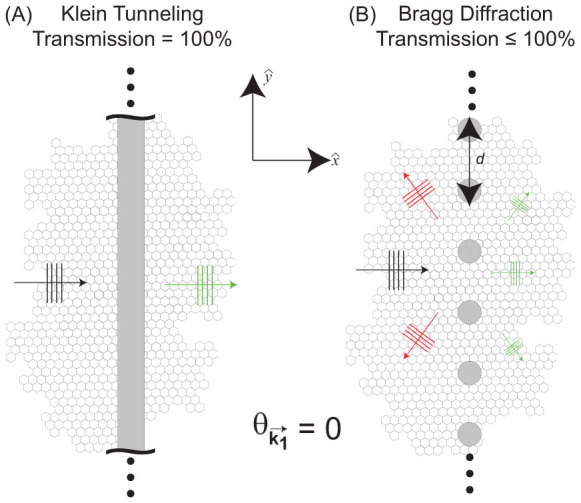
Scattering of Dirac plane wave spinors at normal incidence 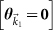 to either (A) a one-dimensional potential barrier or (B) a one-dimensional array of localized cylindrically symmetric scatterers in graphene where the *n^th^* scatterer is centered at 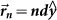 with *d* being the spacing between nearest neighbor scatterers. (A) For a one-dimensional barrier, a normally incident wave is perfectly transmitted as a result of Klein tunneling^19^. (B) For a one-dimensional array of localized scatterers, a normally incident wave can be backscattered into other open scattering channels when 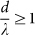 thereby leading to transmission probabilities that can be considerably less than unity.

**Figure 2:**
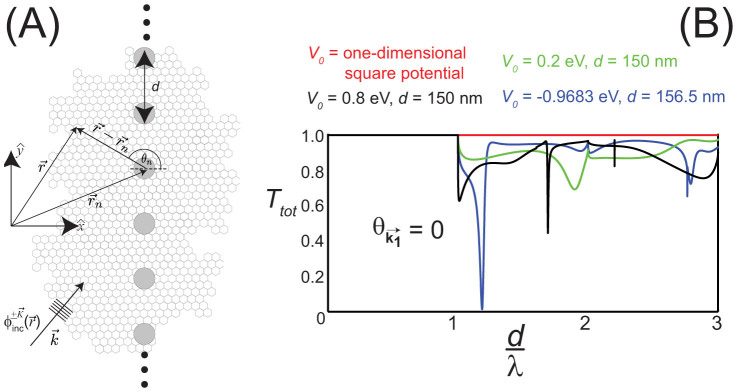
(A) Scattering of an incident Dirac plane wave spinor of energy *E* = 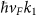 ≥ 0, 
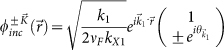
, from a one-dimensional array of localized cylindrically symmetric scatterers in graphene. The unit cell for the scattering array consists of a single scatterer with the position of the *n^th^* scatterer given by 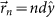. (B) Transmission probability, *T_tot_*, for a plane wave normally incident 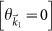 to either a (red line) one-dimensional potential barrier of width 40 nm [*T_tot_* = 1 for all potentials studied in this work] or a one-dimensional array of localized cylindrically symmetric scatterers of radius *r_s_* = 20 nm with the following scattering potentials and lattice spacings: (blue) *V*_0_ = −0.9683 eV and *d* = 156.5 nm, (green) *V*_0_ = 0.2 eV and *d* = 150 nm, and (black) *V*_0_ = 0.8 eV and *d* = 150 nm. For 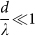, the one-dimensional array of scatterers appear as a uniform one-dimensional potential barrier (black and green) or well (blue) and thus *T_tot_* = 1. When 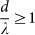, however, the incident electron wave can be reflected into waves with wave vectors 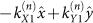 for *n* ≠ 0 that do not correspond to direct backscattering. As a result, *T_tot_* ≤ 1 when 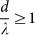.

In Fig. 2, numerical calculations of the total transmission probability, 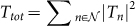, for 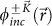 normally incident 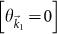 to a one-dimensional array of localized cylindrically symmetric scatterers of radius *r_s_* = 20 nm as a function of 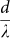 are shown for the following scattering potentials and lattice spacings: (black) *V*_0_ = 0.8 eV and *d* = 150 nm, (green) *V*_0_ = 0.2 eV and *d* = 150 nm, and (blue) *V*_0_ = −0.9683 eV and *d* = 156.5 nm. For reference, *T_tot_* = 1 is represented by a red line, which is the exact transmission probability for a wave normally incident to a constant one-dimensional potential barrier^19^ as shown in Fig. 1(A). For 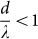, *T_tot_* = 1 for all *V*_0_ since only the *n* = 0 scattering channel is open, i.e., 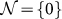. As a result, the incident electron wave is prohibited from direct backscattering due to time-reversal symmetry leading to *T_tot_* = 1. This can also be understood by the fact that when 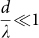, the scattering array effectively appears as a constant one-dimensional potential barrier [Fig. 1(A)] where *T_tot_* = 1 for 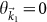. However, when 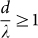, the incident wave can now backscatter into additional open scattering channels, 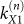 and 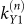 for *n* ≠ 0 in Eq. (3), that do not correspond to direct backscattering, thereby leading to *T_tot_* ≤ 1. Although additional open scattering channels are now available for the incident electron wave to scatter into when 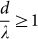 for 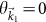, *T_tot_* depends on *V*_0_. For example, *T_tot_* decreased to 0.6277 at 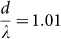 for *V*_0_ = 0.8 eV and *d* = 150 nm [Fig. 2, black curve] and *T_tot_* = 0.0134 at 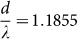 for *V*_0_ = −0, 9683 eV and *d* = 31.3 nm [Fig. 1, blue curve].

**Figure 3:**
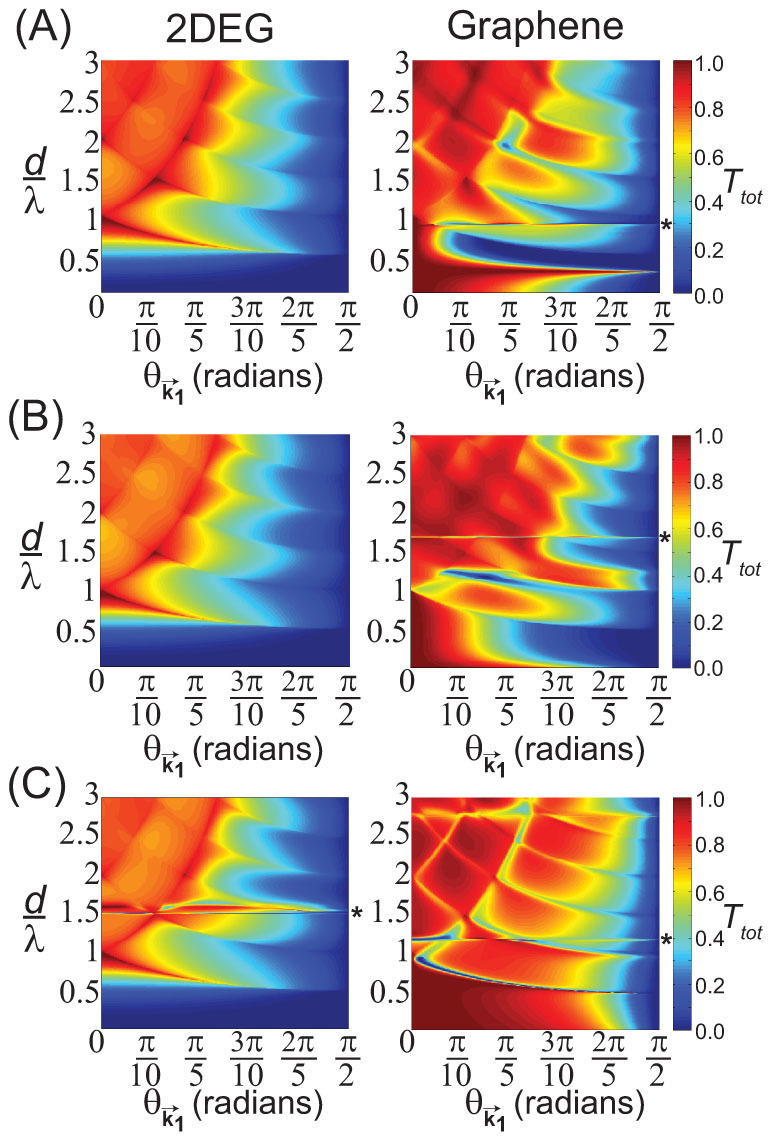
Total transmission probability, *T_tot_*, for a plane wave incident to an infinite one-dimensional array of localized cylindrically symmetric scatterers of radius *r_s_* = 20 nm as a function of incident angle, 
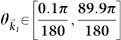
, and the ratio of lattice spacing to wavelength, 
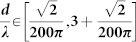
, in a (left) 2DEG and in (right) graphene for following scattering potentials and lattice spacings: (A) *V*_0_ = 0.8 eV [*l_max_* ranging up to *l_max_* = 4 in both graphene and the 2DEG] and *d* = 150 nm, (B) *V*_0_ = 0.2 eV [*l_max_* ranging up to *l_max_* = 4 in both graphene and the 2DEG] and *d* = 150 nm, and (C) *V*_0_ = −0.9683 eV [*l_max_* ranging up to *l_max_* = 6 for graphene and *l_max_* = 4 for the 2DEG] and *d* = 156.5 nm. In Figs. 3(A) and 3(B), the wave vector within the scattering potential was given by 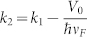 in graphene and −*ik*_2_ in the 2DEG since 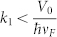 for the range of 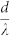 plotted in Figs. 3(A) and 3(B). In Fig. 3(C), the wave vector inside the scattering potential was given by 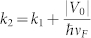 in both graphene and the 2DEG. Asterisks (*) denote those values of 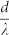 where interference between higher partial waves from the individual scatterers generate sharp features in *T_tot_* [Eq. (4)].

At non-normal incidence, the incident Dirac plane wave can undergo specular reflection and therefore have *T_tot_* < 1. In Figure 3, a comparison of *T_tot_* as a function of 
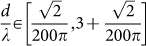
 and incident angle, 
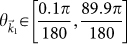
, in graphene is shown [Fig. 3, right]. For comparison, the total transmission probability for non-spinor or achiral plane waves found in a regular two-dimensional electron gas (2DEG) with 
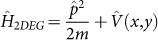
 is also shown [Fig. 3, left]. The same scattering potentials and lattice spacings used in Figure 2 were also used in the calculations shown in Fig. 3: [Fig. 3(A)] *V*_0_ = 0.2 eV and *d* = 150 nm, [Fig. 3(B)] *V*_0_ = 0.8 eV and *d* = 150 nm, and [Fig. 3(C)] *V*_0_ = −0.9683 eV and *d* = 156.5 nm. Further details of the calculations in Fig. 3 are given in Supporting Information. For the 2DEG, *T_tot_* was similar for all scattering potentials studied [Fig. 3(A)–3(C), left], with *T_tot_* → 1 only after 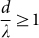 at 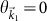. For 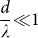, there was negligible transmission of the incident wave in the 2DEG for all 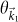. In graphene, however, the dependence of *T_tot_* on 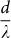 and 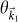 in Fig. 3 (right) was sensitive to *V*_0_. For 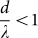 and 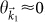, *T_tot_* ≈ 1 in all cases as a result of Klein tunneling as previously illustrated in Fig. 2. However, for 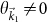, specular reflection can occur leading to *T_tot_* < 1 even for 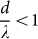. Again, above 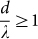, the incident wave can backscatter along the Bragg directions, thereby leading to a reduction in *T_tot_* even at normal incidence.

In Fig. 3, sharp features in *T_tot_* (indicated by * in Fig. 3) were also observed around the following values of 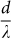 in graphene: [Fig. 3(B), right] 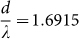 for *V*_0_ = 0.8 eV, [Fig. 3(A), right] 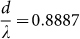 for *V*_0_ = 0.2 eV, and [Fig. 3(C), right] 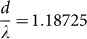 for *V*_0_ = −0.9683 eV. These sharp changes in *T_tot_* appear to result from the interference between partial waves from the individual scatterers at values of *k*_1_*d* where *s_l_* → −1 for at least one of the higher partial waves with *l* ≥ 2 while at the same time |*s*_0_| ∈ [0.8, 1] and/or |*s*_1_| ∈ [0.8, 1]. Approximate values for these *k*_1_*d* where *s_l_* → −1 can be determined from *s_l_* [Eq. (6)] and are solutions to the following equation:
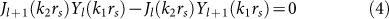
where *Y_l_*(*z*) is a bessel function of the second-kind. In Figure 3, the interference between the *l* = 0 and *l* = 2 partial waves was observed in Fig. 3(A) and Fig. 3(C) at 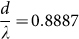 (|*s*_0_| = 0.9355 and |*s*_2_| = 0.9999) and 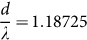 (|*s*_0_| = 0.8863 and |*s*_2_| = 0.9994), respectively, whereas the interference between the *l* = 0, *l* = 1, and *l* = 3 partial waves was observed in Fig. 3(B) at 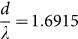 (|*s*_0_| = 0.7964, |*s*_1_| = 0.8005, and |*s*_2_| = 1). Note that a similar interference between higher partial waves was also observed in the 2DEG near 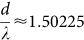 [Fig. 3(C), left] with an attractive scattering potential, *V*_0_ = −0.9683 eV, which was a result of the interference between the *l* = 0 and *l* = 3 partial waves (|*s*_0_| = 0.9436 and |*s*_3_| = 0.8609). Furthermore, calculations of *T_tot_* in the 2DEG using the scattering amplitudes in graphene [*s_l_* in Eq. (6)] also exhibited sharp features in *T_tot_* at the same values of *k*_1_*d* (data not shown). The effects of partial interference between higher partial waves that suppress forward scattering have been previously noted in graphene^20^ and for Mie scattering in optical systems^21^.

## Discussion

A theory for scattering of electron waves incident to a one-dimensional array of localized cylindrically symmetric scatterers [Figure 2(A)] in graphene was used to study the transmission probability through the scattering array as a function of angle of incidence, 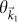, and wavelength *λ* [see Supporting Information for a derivation of the theory]. When the spacing between scatterers, *d*, is much less than 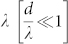, the scattering array in Fig. 2(A) acts like a continuous one-dimensional potential barrier/well [Fig. 1(A)]. In this case, electron waves normally incident to the scattering array are perfectly transmitted as a consequence of Klein tunneling^15^,^19^. However, when 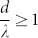, the incident electron waves are able to “resolve” the fact that the scattering array is made up of discrete, localized scatterers that can reflect the incident electron wave along the Bragg directions that do not correspond to direct backscattering [Fig. 1(B)]. As a result, the transmission probabilities can be significantly less than one when 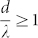, even at normal incidence [Fig. 2]. In effect, placing periodic openings into a constant one-dimensional potential barrier/well can, somewhat counterintuitively, reduce the transmission probability at normal incidence, i.e., suppress Klein tunneling, in graphene. It was demonstrated [Fig. 3, right] that the dependence of the transmission probabilities on incident angle, 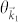, and electron wavelength was more sensitive to the scattering potential in graphene relative to that observed for a regular two-dimensional electron gas (2DEG). Furthermore, when *s_l_*_′_ → −1 for at least one higher partial wave with *l*′ ≥ 2 while |*s_l_*_′_| ≈ |*s*_0_| and/or |*s_l_*_′_| ≈ |*s*_1_|, the interference between the partial waves from the individual scatterers resulted in sharp features in the transmission probabilities [Fig. 3, right]. Similar features were also observed in the transmission probability for a 2DEG with an attractive scattering potential [Fig. 3(C), left]. While only a one-dimensional periodic array of localized scatterers was considered in this work, any potential that has spatial variations larger than the incident electron wavelength will generate non-specular or diffuse reflection that will suppress Klein tunneling. Such potentials could be useful in realizing future graphene electronic devices, such as a graphene field effect transistor^22^. Finally, the results presented in this work could be applied to other physical systems that behave like massless Dirac fermions, such as the surface states of topological insulators^23^,^24^,^25^, optical analogues of graphene^26^, and trapped ions^27^.

## Methods

The theory for scattering of a massless Dirac plane wave spinor from a one-dimensional array of localized cylindrically symmetric scatterers [Fig. 1(B)] is derived in Supporting Information^28^,^29^,^30^,^31^,^32^, where it is shown that the full scattering solution for 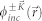 incident to the scattering array shown in Fig. 1(B), 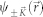, can be written as [for *x* ≠ 0]:
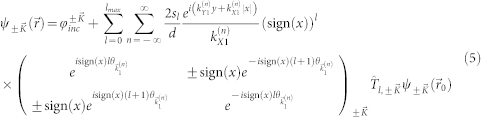
where 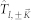 is the single scatterer *l^th^*-partial wave *t*–matrix operator, *l_max_* is the maximum number of partial waves that are included in the calculations, 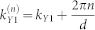 for integer *n*, and either 
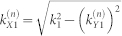
 and 
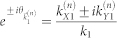
 for 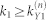 or 
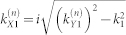
 and 
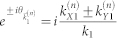
 for 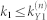. In Eq. (5), 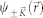 consists of a series of plane waves 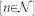 that are either transmitted [*x* > 0] or reflected [*x* < 0] from the scattering array along with an infinite number of waves 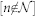 that are evanescent along the 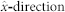 and freely propagating along the 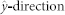. These evanescent waves are a consequence of the periodicity of the one-dimensional array of scatterers. The transmission [*T_n_* in Eq. (2) for 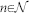] and reflection [*R_n_* in Eq. (3) for 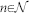] coefficients can be determined from Eq. (5), and explicit expressions for *T_n_* and *R_n_* are given in Supporting Information.

In all simulations, each scatterer was modeled as a cylindrically symmetric barrier/well of potential *V*_0_ and radius *r_s_*. For an individual scatterer, the *l^th^* partial wave scattering amplitude is given by^28^,^33^:
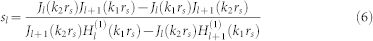
where 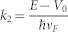, and *J_l_*(*z*) and 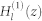 are first-order bessel and hankel functions of order *l*, respectively. The maximum partial wave used in the calculations, *l_max_*, was chosen to take into account 99.9% of the total scattering amplitude for an individual scatterer, i.e., 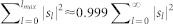. For the calculations of *T_tot_* in a 2DEG [Fig. 3 (left)], previous work^32^ on scattering from one-dimensional periodic grating in a 2DEG was generalized to include higher partial waves. Details of these calculations are also given in Supporting Information.

## Author Contributions

J.D.W. performed theoretical and numerical calculations and wrote manuscript. D.H. worked on initial simulations for the project and helped in manuscript preparation.

## Supplementary Material

Supplementary Information Supporting Information for
