# Supplementary material for: Suppressing Klein tunneling in graphene using a one-dimensional array of localized scatterers
Source: Sci Rep. 2015 Feb 13;5:8435. doi: 10.1038/srep08435 (PMC4327422; doi:10.1038/srep08435)
Supplement: Supplementary Information — Supporting Information for [file srep08435-s1.pdf]

Supporting Information for "Suppressing Klein tunneling in graphene using a one-dimensional array of localized scatterers"

Jamie D. Walls[1,*] and Daniel Hadad[1]

[1]*Department of Chemistry, University of Miami, Coral Gables, Florida 33124,USA*

* Corresponding author: jwalls@miami.edu

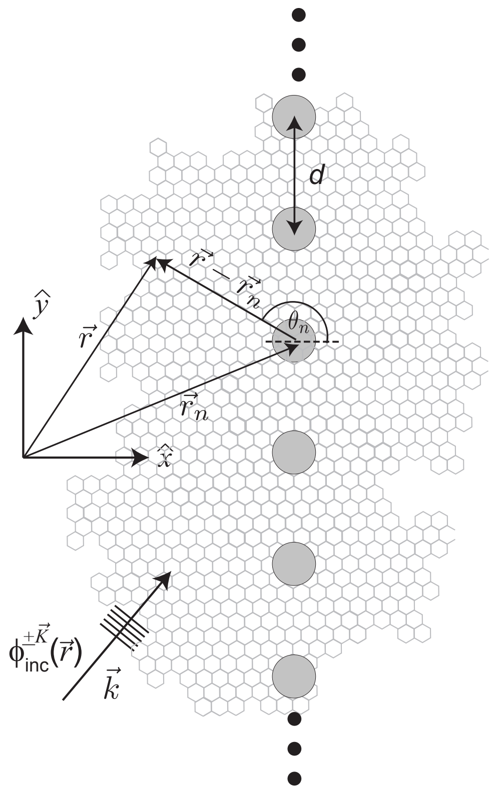

FIG. 1. Scattering of an incident Dirac plane wave spinor of energy $E = \hbar v_F k_1 \geq 0$, $\phi^{\pm\vec{K}}_{inc}(\vec{r}) = \sqrt{\frac{k_1}{2v_F k_{X1}}} e^{i\vec{k}_1\cdot\vec{r}} \begin{pmatrix} 1 \\ \pm e^{i\theta_{\vec{k}_1}} \end{pmatrix}$, from a one-dimensional array of localized cylindrically symmetric scatterers in graphene. The unit cell for the scattering array consists of a single scatterer with the position of the nth scatterer given by $\vec{r}_n = nd\hat{y}$.

In the following, the basic theory for intravalley multiple scattering in graphene is presented and applied to the problem of a plane wave scattering from an infinite, one-dimensional array of localized scatterers. First, the basic formalism for calculating the scattering solutions for a plane wave scattered from a finite number of localized scatterers in graphene is presented. Next, the scattering wave functions, $\psi_{\pm\vec{K}}(\vec{r})$, for the single and two scatterer cases are explicitly presented. The theory is then extended to the case of scattering from an infinite, one-dimensional array of localized scatterers in graphene where explicit expressions for the reflection and transmission coefficients are provided. Finally, the analogous theory for scattering from an infinite, one-dimensional array of localized scatterers in a two-dimensional electron gas (2DEG) is derived for comparison to the graphene results.

I. INTRAVALLEY MULTIPLE SCATTERING FORMALISM

In the following, we briefly review the basic formalism for intravalley multiple scattering in graphene[1], where the scattering solutions are expanded about either the $+\vec{K}$ or $-\vec{K}$ Dirac points, where $\vec{K} = \frac{4\pi\sqrt{3}}{9b}\hat{x}$ and $b = 1.42\AA$ is the carbon-carbon bond length in graphene. Let $\phi^{\pm\vec{K}}_{inc}(\vec{r})$ be an

incident Dirac plane wave spinor of energy $E = \hbar v_F k_1 = \frac{h v_F}{\lambda}$ normalized to unit flux along the $\widehat{x}$-direction, $\phi_{inc}^{\vec{K}}(\vec{r}) = \sqrt{\frac{k_1}{2 v_F k_{X1}}} e^{\vec{k}_1 \cdot \vec{r}} \begin{pmatrix} 1 \\ e^{i\theta_{\vec{k}_1}} \end{pmatrix}_{\vec{K}}$ or $\phi_{inc}^{-\vec{K}}(\vec{r}) = \sqrt{\frac{k_1}{2 v_F k_{X1}}} e^{\vec{k}_1 \cdot \vec{r}} \begin{pmatrix} 1 \\ -e^{i\theta_{\vec{k}_1}} \end{pmatrix}_{-\vec{K}}$ with a wave vector given by $\vec{k}_1 = k_1 \cos(\theta_{\vec{k}_1}) \widehat{x} + k_1 \sin(\theta_{\vec{k}_1}) \widehat{y} = k_{X1} \widehat{x} + k_{Y1} \widehat{y}$ for $\theta_{\vec{k}_1} \in \left(-\frac{\pi}{2}, \frac{\pi}{2}\right)$, and $\lambda = \frac{2\pi}{k_1}$ is the wavelength of the incident wave. In all calculations, $\hbar v_F = 1.0558 \times 10^{-28}$ J-m. In the following, a scattering potential consisting of a linear arrangement of $2N+1$ identical scatterers along the $\widehat{y}$-direction is considered with the scatterers' positions indexed by the integer $n \in [-N, N]$, $\vec{r}_n = nd\widehat{y}$ where d is the spacing between adjacent scatterers [Fig. 1].

The total wave function for a Dirac plane wave spinor incident to the linear array of $2N+1$ scatterers, $\psi_{\pm\vec{K}}(\vec{r})$, can be written as:

$$\psi_{\pm\vec{K}}(\vec{r}) = \phi_{inc}^{\pm\vec{K}}(\vec{r}) + \sum_{l=0}^{l_{max}} \sum_{n=-N}^{N} \frac{4i\hbar v_F}{k_1} s_l \widehat{G}_{l,\pm\vec{K}}(\vec{r}, \vec{r}_n, E) \widehat{T}_{l,\pm\vec{K}} \psi_{\pm\vec{K}}(\vec{r}_n) \tag{1}$$

where s_l is the scattering amplitude of the lth partial wave, $l_{max}+1$ are the number of partial waves that are included in calculation of $\psi_{\pm\vec{K}}(\vec{r})$ in Eq. (1) with $l_{max} \geq 0$, $\widehat{T}_{l,\pm\vec{K}} \psi_{\pm\vec{K}}(\vec{r}_n) \equiv \widehat{T}_{l,\pm\vec{K}} \left(\psi_{\pm\vec{K}}(\vec{r})\right)_{\vec{r}=\vec{r}_n}$, and

$$\begin{aligned} \widehat{G}_{l,\pm\vec{K}}(\vec{r}, \vec{r}_n, E) &= -\frac{i^{l+1} k_1}{4\hbar v_F} \begin{pmatrix} H_l^{(1)}(k_1\rho_n) e^{il\theta_n} & \pm i H_{l+1}^{(1)}(k_1\rho_n) e^{-i(l+1)\theta_n} \\ \pm i H_{l+1}^{(1)}(k_1\rho_n) e^{i(l+1)\theta_n} & H_l^{(1)}(k_1\rho_n) e^{-il\theta_n} \end{pmatrix}_{\pm\vec{K}} \\ &= -\frac{ik_1}{4\hbar v_F} \begin{pmatrix} \hat{L}_+^l [H_0^{(1)}(k_1\rho_n)] & \pm \hat{L}_-^{l+1} [H_0^{(1)}(k_1\rho_n)] \\ \pm \hat{L}_+^{l+1} [H_0^{(1)}(k_1\rho_n)] & \hat{L}_-^l [H_0^{(1)}(k_1\rho_n)] \end{pmatrix}_{\pm\vec{K}} \end{aligned} \tag{2}$$

where $H_l^{(1)}(z)$ is a hankel function of order l, $\rho_n = |\vec{r} - \vec{r}_n|$, $e^{\pm i\theta_n} = \frac{(\vec{r}-\vec{r}_n)\cdot(\widehat{x} \pm i\widehat{y})}{\rho_n}$, and $\widehat{T}_{l,\pm\vec{K}}$ is the l-partial wave $t-$matrix operator given by:

$$\widehat{T}_{l,\pm\vec{K}} = \begin{pmatrix} \hat{L}_-^l & 0 \\ 0 & \hat{L}_+^l \end{pmatrix}_{\pm\vec{K}} \tag{3}$$

where $\hat{L}_\pm = \frac{1}{ik_1} e^{\pm i\theta} \left(\frac{\partial}{\partial_r} \pm \frac{i}{r} \frac{\partial}{\partial_\theta}\right) = \frac{1}{ik_1} \left(\frac{\partial}{\partial_x} \pm i \frac{\partial}{\partial_y}\right)$, and $\widehat{T}_{0,\pm\vec{K}} = \widehat{1}$ is the 2×2 identity matrix. In writing Eq. (1), the scattering amplitudes were taken to satisfy the relationship $s_l = s_{-(l+1)}$, which is a consequence of the scattering potential being identical over both inequivalent lattice sites in graphene. For the numerical calculations performed in this work, each scatterer was modeled as a cylindrically symmetric step potential with an effective radius of r_s, i.e., the potential for the nth scatterer was given by $V_0 \Theta(\vec{r} - \vec{r}_n)$ where $\Theta(\vec{r} - \vec{r}_n) = 1$ if $|\vec{r} - \vec{r}_n| \leq r_s$ and $\Theta(\vec{r} - \vec{r}_n) = 0$

for $|\vec{r}-\vec{r}_n| > r_s$. To consider only *intravalley* scattering and to neglect *intervalley* scattering, $r_s \gg b = 1.42\AA$. For an individual scatterer, the lth partial wave scattering amplitude is given[1, 2] by:

$$s_l = \frac{J_l(k_2 r_s)J_{l+1}(k_1 r_s) - J_l(k_1 r_s)J_{l+1}(k_2 r_s)}{J_{l+1}(k_2 r_s)H_l^{(1)}(k_1 r_s) - J_l(k_2 r_s)H_{l+1}^{(1)}(k_1 r_s)} \tag{4}$$

where $k_2 = \frac{E-V_0}{\hbar v_F}$, and $J_l(z)$ is a bessel function of the first kind of order l, respectively. In all simulations, l_{max} was chosen to take into account 99.9% of the total scattering amplitude for an individual scatterer, i.e., $\sum_{l=0}^{l_{max}} |s_l|^2 \approx 0.999 \sum_{l=0}^{\infty} |s_l|^2$.

Knowledge of $\widehat{T}_{l,\pm\vec{K}}\psi_{\pm\vec{K}}(\vec{r}_n)$ for all n and $l \in [0, l_{max}]$ completely determines $\psi_{\pm\vec{K}}(\vec{r})$ in Eq. (1); these can be determined self-consistently as follows:

$$\widehat{T}_{l',\pm\vec{K}}\psi_{\pm\vec{K}}(\vec{r}_m) = \widehat{T}_{l',\pm\vec{K}}\phi_{inc}^{\pm\vec{K}}(\vec{r}_m) + \sum_{l=0}^{l_{max}}\sum_{n\neq m} s_l T_{l',\pm\vec{K}}\left[\widehat{G}_{l,\pm\vec{K}}(\vec{r}_m,\vec{r}_n,E)\right]\widehat{T}_{l,\pm\vec{K}}\psi_{\pm\vec{K}}(\vec{r}_n) \tag{5}$$

where

$$\widehat{T}_{l',\pm\vec{K}}\left[\widehat{G}_{l,\pm\vec{K}}(\vec{r}_n,\vec{r}_j,E)\right] = -\frac{ik_1}{4\hbar v_F} i^{l+l'} \begin{pmatrix} (-1)^{l'}H_{l-l'}^{(1)}(k_1 r_{nj})e^{i(l-l')\theta_{nj}} & \pm iH_{l+l'+1}^{(1)}(k_1 r_{nj})e^{-i\theta_{nj}(l+l'+1)} \\ \pm iH_{l+l'+1}^{(1)}(k_1 r_{nj})e^{i\theta_{nj}(l+l'+1)} & (-1)^l H_{l'-l}^{(1)}(k_1 r_{nj})e^{i\theta_{nj}(l'-l)} \end{pmatrix}_{\pm\vec{K}} \tag{6}$$

and $r_{nj} = |\vec{r}_n - \vec{r}_j|$ and $e^{\pm i\theta_{nj}} = \frac{(\vec{r}_n-\vec{r}_j)\cdot(\widehat{x}\pm i\widehat{y})}{r_{nj}}$. In this case, Eq. (5) formally represents a set of $2(2N+1)\times(l_{max}+1)$ equations that can be solved self-consistently as follows[1]:

Define the following $2(l_{max}+1)\times 1$ column vectors related to the total and incident wave functions evaluated at scatterer $j \in [-N, -N+1, \ldots, N-1, N]$:

$$\widehat{\widehat{T}}\widehat{\psi}_{\pm\vec{K}}(\vec{r}_j) = \begin{pmatrix} \widehat{T}_{0,\pm\vec{K}}\psi_{\pm\vec{K}}(\vec{r}_j) \\ \widehat{T}_{1,\pm\vec{K}}\psi_{\pm\vec{K}}(\vec{r}_j) \\ \widehat{T}_{2,\pm\vec{K}}\psi_{\pm\vec{K}}(\vec{r}_j) \\ \vdots \\ \widehat{T}_{l_{max},\pm\vec{K}}\psi_{\pm\vec{K}}(\vec{r}_j) \end{pmatrix}, \widehat{\widehat{T}}\widehat{\phi}_{inc,\pm\vec{K}}(\vec{r}_j) = \begin{pmatrix} \widehat{T}_{0,\pm\vec{K}}\phi_{inc}^{\pm\vec{K}}(\vec{r}_j) \\ \widehat{T}_{1,\pm\vec{K}}\phi_{inc}^{\pm\vec{K}}(\vec{r}_j) \\ \widehat{T}_{2,\pm\vec{K}}\phi_{inc}^{\pm\vec{K}}(\vec{r}_j) \\ \vdots \\ \widehat{T}_{l_{max},\pm\vec{K}}\phi_{inc}^{\pm\vec{K}}(\vec{r}_j) \end{pmatrix} \tag{7}$$

and the following $2(2N+1)(l_{max}+1)\times 1$ column vectors:

$$\widehat{\widehat{\mathbf{T}}}\widehat{\widehat{\psi}}_{\pm\vec{K}} = \begin{pmatrix} \widehat{\widehat{T}}\widehat{\psi}_{\pm\vec{K}}(\vec{r}_N) \\ \widehat{\widehat{T}}\widehat{\psi}_{\pm\vec{K}}(\vec{r}_{N-1}) \\ \vdots \\ \widehat{\widehat{T}}\widehat{\psi}_{\pm\vec{K}}(\vec{r}_0) \\ \vdots \\ \widehat{\widehat{T}}\widehat{\psi}_{\pm\vec{K}}(\vec{r}_{-(N-1)}) \\ \widehat{\widehat{T}}\widehat{\psi}_{\pm\vec{K}}(\vec{r}_{-N}) \end{pmatrix}, \widehat{\widehat{\mathbf{T}}}\widehat{\widehat{\phi}}_{inc,\pm\vec{K}} = \begin{pmatrix} \widehat{\widehat{T}}\widehat{\phi}_{inc,\pm\vec{K}}(\vec{r}_N) \\ \widehat{\widehat{T}}\widehat{\phi}_{inc,\pm\vec{K}}(\vec{r}_{N-1}) \\ \vdots \\ \widehat{\widehat{T}}\widehat{\phi}_{inc,\pm\vec{K}}(\vec{r}_0) \\ \vdots \\ \widehat{\widehat{T}}\widehat{\phi}_{inc,\pm\vec{K}}(\vec{r}_{-(N-1)}) \\ \widehat{\widehat{T}}\widehat{\phi}_{inc,\pm\vec{K}}(\vec{r}_{-N}) \end{pmatrix} \tag{8}$$

Using Eqs. (7) and (8), Eq. (5) for $n=-N$ to $n=N$ and for $l'=0$ to $l'=l_{max}$ can be written compactly as:

$$\widehat{\widehat{\mathbf{T}}}\widehat{\widehat{\psi}}_{\pm\vec{K}} = \left(\widehat{\widehat{\mathbf{1}}} - \widehat{\widehat{\mathbf{TT}}}\right)^{-1} \widehat{\widehat{\mathbf{T}}}\widehat{\widehat{\phi}}_{inc,\pm\vec{K}} \tag{9}$$

where $\widehat{\widehat{\mathbf{1}}}$ is the $2(2N+1)(l_{max}+1)\times 2(2N+1)(l_{max}+1)$ identity matrix, and $\widehat{\widehat{\mathbf{TT}}}$ is a $2(2N+1)(l_{max}+1)\times 2(2N+1)(l_{max}+1)$ matrix given by:

$$\widehat{\widehat{\mathbf{TT}}} = \begin{pmatrix} \widehat{\mathbf{0}} & \widehat{\widehat{TT}}_{\pm\vec{K}}(\vec{r}_N,\vec{r}_{N-1}) & \widehat{\widehat{TT}}_{\pm\vec{K}}(\vec{r}_N,\vec{r}_{N-2}) & \dots & \widehat{\widehat{TT}}_{\pm\vec{K}}(\vec{r}_N,\vec{r}_{-N}) \\ \widehat{\widehat{TT}}_{\pm\vec{K}}(\vec{r}_{N-1},\vec{r}_N) & \widehat{\mathbf{0}} & \widehat{\widehat{TT}}_{\pm\vec{K}}(\vec{r}_{N-1},\vec{r}_{N-2}) & \dots & \widehat{\widehat{TT}}_{\pm\vec{K}}(\vec{r}_{N-1},\vec{r}_{-N}) \\ \vdots & \vdots & \vdots & \ddots & \vdots \\ \widehat{\widehat{TT}}_{\pm\vec{K}}(\vec{r}_{-N},\vec{r}_N) & \widehat{\widehat{TT}}_{\pm\vec{K}}(\vec{r}_{-N},\vec{r}_{N-1}) & \widehat{\widehat{TT}}_{\pm\vec{K}}(\vec{r}_{-N},\vec{r}_{N-2}) & \dots & \widehat{\mathbf{0}} \end{pmatrix} \tag{10}$$

where

$$\widehat{\widehat{TT}}_{\pm\vec{K}}(\vec{r}_n,\vec{r}_j) = \begin{pmatrix} \widehat{G}_{0,\pm\vec{K}}(\vec{r}_n,\vec{r}_j,E) & \widehat{G}_{1,\pm\vec{K}}(\vec{r}_n,\vec{r}_j,E) & \widehat{G}_{2,\pm\vec{K}}(\vec{r}_n,\vec{r}_j,E) & \dots & \widehat{G}_{l_{max},\pm\vec{K}}(\vec{r}_n,\vec{r}_j,E) \\ \widehat{T}_{1,\pm\vec{K}}\left[\widehat{G}_{0,\pm\vec{K}}(\vec{r}_n,\vec{r}_j,E)\right] & \widehat{T}_{1,\pm\vec{K}}\left[\widehat{G}_{1,\pm\vec{K}}(\vec{r}_n,\vec{r}_j,E)\right] & \widehat{T}_{1,\pm\vec{K}}\left[\widehat{G}_{2,\pm\vec{K}}(\vec{r}_n,\vec{r}_j,E)\right] & \dots & \widehat{T}_{1,\pm\vec{K}}\left[\widehat{G}_{l_{max},\pm\vec{K}}(\vec{r}_n,\vec{r}_j,E)\right] \\ \widehat{T}_{2,\pm\vec{K}}\left[\widehat{G}_{0,\pm\vec{K}}(\vec{r}_n,\vec{r}_j,E)\right] & \widehat{T}_{2,\pm\vec{K}}\left[\widehat{G}_{1,\pm\vec{K}}(\vec{r}_n,\vec{r}_j,E)\right] & \widehat{T}_{2,\pm\vec{K}}\left[\widehat{G}_{2,\pm\vec{K}}(\vec{r}_n,\vec{r}_j,E)\right] & \dots & \widehat{T}_{2,\pm\vec{K}}\left[\widehat{G}_{l_{max},\pm\vec{K}}(\vec{r}_n,\vec{r}_j,E)\right] \\ \vdots & \vdots & \vdots & \ddots & \vdots \\ \widehat{T}_{l_{max},\pm\vec{K}}\left[\widehat{G}_{0,\pm\vec{K}}(\vec{r}_n,\vec{r}_j,E)\right] & \widehat{T}_{l_{max},\pm\vec{K}}\left[\widehat{G}_{1,\pm\vec{K}}(\vec{r}_n,\vec{r}_j,E)\right] & \widehat{T}_{l_{max},\pm\vec{K}}\left[\widehat{G}_{2,\pm\vec{K}}(\vec{r}_n,\vec{r}_jE)\right] & \dots & \widehat{T}_{l_{max},\pm\vec{K}}\left[\widehat{G}_{l_{max},\pm\vec{K}}(\vec{r}_n,\vec{r}_j,E)\right] \end{pmatrix} \widehat{\mathbf{S}}_{l_{max}} \tag{11}$$

and

$$\widehat{\mathbf{S}}_{l_{max}} = \frac{4i\hbar v_F}{k_1} \begin{pmatrix} s_0 & 0 & 0 & 0 & 0 & 0 & \dots & 0 & 0 \\ 0 & s_0 & 0 & 0 & 0 & 0 & \dots & 0 & 0 \\ 0 & 0 & s_1 & 0 & 0 & 0 & \dots & 0 & 0 \\ 0 & 0 & 0 & s_1 & 0 & 0 & \dots & 0 & 0 \\ 0 & 0 & 0 & 0 & s_2 & 0 & \dots & 0 & 0 \\ 0 & 0 & 0 & 0 & 0 & s_2 & \dots & 0 & 0 \\ \vdots & \vdots & \vdots & \vdots & \vdots & \vdots & \ddots & \vdots & \vdots \\ 0 & 0 & 0 & 0 & 0 & 0 & \dots & s_{l_{max}} & 0 \\ 0 & 0 & 0 & 0 & 0 & 0 & \dots & 0 & s_{l_{max}} \end{pmatrix} \tag{12}$$

Using Eq. (8) and Eq. (10), $\psi_{\pm\vec{K}}(\vec{r})$ in Eq. (1) can be written compactly as:

$$\begin{aligned} \psi_{\pm\vec{K}}(\vec{r}) &= \phi_{inc}^{\pm\vec{K}}(\vec{r}) + \widehat{\widehat{\mathbf{GG}}}_{\pm\vec{K}}(\vec{r})\widehat{\widehat{\mathbf{T}}}\widehat{\widehat{\psi}}_{\pm\vec{K}} \\ &= \phi_{inc}^{\pm\vec{K}}(\vec{r}) + \widehat{\widehat{\mathbf{GG}}}_{\pm\vec{K}}(\vec{r})\left(\widehat{\widehat{\mathbf{1}}} - \widehat{\widehat{\mathbf{TT}}}\right)^{-1} \widehat{\widehat{\mathbf{T}}}\widehat{\widehat{\phi}}_{inc,\pm\vec{K}} \end{aligned} \tag{13}$$

where $\widehat{\widehat{\mathbf{GG}}}_{\pm\vec{K}}(\vec{r})$ is a $2\times 2(2N+1)(l_{max}+1)$ matrix, $\widehat{\widehat{\mathbf{GG}}}_{\pm\vec{K}}(\vec{r}) = \left[\widehat{\widehat{GG}}(\vec{r},\vec{r}_N)\ \widehat{\widehat{GG}}(\vec{r},\vec{r}_{N-1})\ \ldots \widehat{\widehat{GG}}(\vec{r},\vec{r}_{-N})\right]$ where

$$\widehat{\widehat{GG}}(\vec{r},\vec{r}_j) = \frac{4i\hbar v_F}{k_1}\left[\ s_0\widehat{G}_{0,\pm\vec{K}}(\vec{r},\vec{r}_j,E)\ \ s_1\widehat{G}_{1,\pm\vec{K}}(\vec{r},\vec{r}_j,E)\ \ \ldots\ \ s_{l_{max}}\widehat{G}_{l_{max},\pm\vec{K}}(\vec{r},\vec{r}_j,E)\ \right] \quad (14)$$

II. SCATTERING FROM A SINGLE SCATTERER

We will first consider the problem of scattering from a single scatter located at $\vec{r}_0 = 0$. In this case, Eq. (5) becomes $\widehat{T}_{l',\pm\vec{K}}\psi_{\pm\vec{K}}(0) = \widehat{T}_{l',\pm\vec{K}}\phi_{inc}^{\pm\vec{K}}(0)$, which gives:

$$\widehat{T}_{l',\pm\vec{K}}\psi_{\pm\vec{K}}(0) = \sqrt{\frac{k_1}{2v_F k_{X1}}}\begin{pmatrix} e^{-il'\theta_{\vec{k}_1}} \\ e^{i(l'+1)\theta_{\vec{k}_1}} \end{pmatrix} \quad (15)$$

Inserting Eq. (15) into Eq. (1) gives:

$$\begin{aligned}\psi_{\pm\vec{K}}(\vec{r}) &= \phi_{inc}^{\pm\vec{K}}(\vec{r}) + \sum_{l=0}^{l_{max}}\frac{4i\hbar v_F}{k_1}s_l\widehat{G}_{l,\pm\vec{K}}(\vec{r},0,E)\widehat{T}_{l,\pm\vec{K}}\psi_{\pm\vec{K}}(0) \\ &= \phi_{inc}^{\pm\vec{K}}(\vec{r}) + \sum_{l=0}^{l_{max}} i^l s_l\sqrt{\frac{k_1}{2v_F k_{X1}}}\begin{pmatrix} H_l^{(1)}(k_1 r)e^{il(\theta_0-\theta_{\vec{k}_1})} + iH_{l+1}(k_1 r)e^{-i(l+1)(\theta_0-\theta_{\vec{k}_1})} \\ \pm e^{i\theta_{\vec{k}_1}}\left(H_l^{(1)}(k_1 r)e^{-il(\theta_0-\theta_{\vec{k}_1})} + iH_{l+1}^{(1)}(k_1 r)e^{i(l+1)(\theta_0-\theta_{\vec{k}_1})}\right)\end{pmatrix}\end{aligned} \quad (16)$$

where $r = |\vec{r}-\vec{r}_0|$. For $k_1 r \gg 1$, Eq. (16) can be approximated by:

$$\psi_{\pm\vec{K}}(\vec{r}) = \phi_{inc}^{\pm\vec{K}}(\vec{r}) + \sum_{l=0}^{l_{max}}\frac{2s_l e^{-i\frac{\pi}{4}}\cos\left(\left(l+\frac{1}{2}\right)\left(\theta_0-\theta_{\vec{k}_1}\right)\right)}{\sqrt{\pi v_F k_{X1} r}}\begin{pmatrix} e^{-i\frac{\theta_0-\theta_{\vec{k}_1}}{2}} \\ \pm e^{i\frac{\theta_0-\theta_{\vec{k}_1}}{2}}\end{pmatrix} \quad (17)$$

As $k_1 r \to \infty$, the scattered wave function asymptotically decays to zero as $r^{-\frac{1}{2}}$ leaving only the initial plane wave, $\psi_{\pm\vec{K}}(\vec{r}) \approx \phi_{inc}^{\pm\vec{K}}(\vec{r})$.

III. SCATTERING FROM TWO IDENTICAL SCATTERERS

Consider two identical scatterers, one located at $\vec{r}_0 = 0$ and the other at $\vec{r}_1 = d\hat{y}$. In this case, Eq. (1) becomes:

$$\begin{aligned}\psi_{\pm\vec{K}}(\vec{r}) &= \phi_{inc}^{\pm\vec{K}}(\vec{r}) + \sum_{l=0}^{l_{max}}\frac{4i\hbar v_F}{k_1}s_l\left[\widehat{G}_{l,\pm\vec{K}}(\vec{r},\vec{r}_0,E)\widehat{T}_{l,\pm\vec{K}}\psi_{\pm\vec{K}}(\vec{r}_0) + \widehat{G}_{l,\pm\vec{K}}(\vec{r},\vec{r}_1,E)\widehat{T}_{l,\pm\vec{K}}\psi_{\pm\vec{K}}(\vec{r}_1)\right] \\ &= \phi_{inc}^{\pm\vec{K}}(\vec{r}) + \widehat{\widehat{\mathbf{GG}}}_{\pm\vec{K}}(\vec{r})\left(\widehat{\widehat{\mathbf{1}}} - \widehat{\widehat{\mathbf{TT}}}\right)^{-1}\widehat{\widehat{\mathbf{T}}}\widehat{\widehat{\phi}}_{inc,\pm\vec{K}}\end{aligned} \quad (18)$$

where

$$\widehat{\widehat{\mathbf{TT}}} = \begin{pmatrix} \widehat{\mathbf{0}} & \widehat{\widehat{TT}}_{\pm\vec{K}}(\vec{r}_1,\vec{r}_0) \\ \widehat{\widehat{TT}}_{\pm\vec{K}}(\vec{r}_0,\vec{r}_1) & \widehat{\mathbf{0}} \end{pmatrix}$$

$$\widehat{\widehat{\mathbf{T}}}\widehat{\boldsymbol{\phi}}_{inc,\pm\vec{K}} = \begin{pmatrix} e^{ik_{Y1}d}\widehat{\widehat{T}}\widehat{\phi}_{inc,\pm\vec{K}}(\vec{r}_0) \\ \widehat{\widehat{T}}\widehat{\phi}_{inc,\pm\vec{K}}(\vec{r}_0) \end{pmatrix}$$

$$\widehat{\widehat{T}}\widehat{\phi}_{inc,\pm\vec{K}}(\vec{r}_0) = \sqrt{\frac{k_1}{2v_F k_{X1}}} \begin{pmatrix} 1 \\ \pm e^{i\theta_{\vec{k}_1}} \\ e^{-i\theta_{\vec{k}_1}} \\ \pm e^{2i\theta_{\vec{k}_1}} \\ e^{-i2\theta_{\vec{k}_1}} \\ \pm e^{3i\theta_{\vec{k}_1}} \\ \vdots \\ e^{-il_{max}\theta_{\vec{k}_1}} \\ \pm e^{i(l_{max}+1)\theta_{\vec{k}_1}} \end{pmatrix}$$

$$\widehat{\widehat{\mathbf{GG}}}_{\pm\vec{K}}(\vec{r}) = \left[\widehat{\widehat{GG}}(\vec{r},\vec{r}_1)\ \widehat{\widehat{GG}}(\vec{r},\vec{r}_0)\right] \tag{19}$$

Formally, inverting $\widehat{\widehat{\mathbf{1}}} - \widehat{\widehat{\mathbf{TT}}}$ requires finding the roots to a polynomial of order $4(l_{max}+1)$, which must be numerically solved when $l_{max} > 0$. As in the single scattering case, the scattered wave function in Eq. (18) scales as $r^{-\frac{1}{2}}$ for $k_1\rho_1 \approx k_1 r \gg 1$ and $k_1\rho_0 \approx k_1 r \gg 1$, which gives $\psi_{\pm\vec{K}}(\vec{r}) \approx \phi_{inc}^{\pm\vec{K}}(\vec{r})$ for $k_1 r \gg 1$.

IV. SCATTERING FROM AN INFINITE ONE-DIMENSIONAL ARRAY OF SCATTERERS

In the following, the theory for the scattering of plane waves from an infinite, periodic one-dimensional array of identical scatterers ($N \to \infty$) with lattice constant d [Figure 1] is presented. From translational symmetry, the total wave function satisfies the relation that $\psi_{\pm\vec{K}}(\vec{r}+nd\widehat{y}) = \psi_{\pm\vec{K}}(\vec{r})e^{ik_{Y1}nd}$, which means that only $\psi_{\pm\vec{K}}(\vec{r})$ between $-\frac{d}{2} \le y \le \frac{d}{2}$ needs to be calculated. To calculate $\psi_{\pm\vec{K}}(\vec{r})$ in Eq. (1), one can use the fact that translational symmetry implies that $\widehat{T}_{l,\pm\vec{K}}\psi_{\pm\vec{K}}(\vec{r}_n) = e^{ik_{Y1}nd}\widehat{T}_{l,\pm\vec{K}}\psi_{\pm\vec{K}}(\vec{r}_0)$, in which case the total wave function in Eq. (1) can be written as:

$$\psi_{\pm\vec{K}}(\vec{r}) = \phi_{inc}^{\pm\vec{K}}(\vec{r}) + \sum_{l=0}^{l_{max}}\sum_{n=-\infty}^{\infty}\frac{4i\hbar v_F}{k_1}s_l\widehat{G}_{l,\pm\vec{K}}(\vec{r},nd\widehat{y},E)e^{indk_{Y1}}\widehat{T}_{l,\pm\vec{K}}\psi_{\pm\vec{K}}(\vec{r}_0) \tag{20}$$

The various $\widehat{T}_{l,\pm\vec{K}}\psi_{\pm K}(\vec{r}_0)$ in Eq. (20) are determined self-consistently by:

$$\widehat{T}_{l',\pm\vec{K}}\psi_{\pm\vec{K}}(\vec{r}_0) = \widehat{T}_{l',\pm\vec{K}}\phi_{inc}^{\pm\vec{K}}(\vec{r}_0) + \sum_{l=0}^{l_{max}}\sum_{n\neq 0} s_l\left[\widehat{T}_{l',\pm\vec{K}}\widehat{G}_{l,\pm\vec{K}}(\vec{r}_0,\vec{r}_n,E)\right]e^{ink_{Y1}d}\widehat{T}_{l,\pm\vec{K}}\psi_{\pm\vec{K}}(\vec{r}_0) \tag{21}$$

Eq. (21) gives a total of $2(l_{max}+1)$ sets of equations, which can be written as:

$$\left(\widehat{\widehat{\mathbf{1}}} - \widehat{\widehat{\mathbf{TG}}}\right)\widehat{\widehat{\mathbf{T}}}\widehat{\psi}_{\pm\vec{K}}(\vec{r}_0) = \widehat{\widehat{\mathbf{T}}}\widehat{\phi}_{inc}^{\pm\vec{K}}(\vec{r}_0) \tag{22}$$

where $\widehat{\widehat{\mathbf{1}}}$ is a $2(l_{max}+1)\times 2(l_{max}+1)$ identity matrix, $\widehat{\widehat{\mathbf{T}}}\widehat{\psi}_{\pm\vec{K}}(\vec{r}_0)$ and $\widehat{\widehat{\mathbf{T}}}\widehat{\phi}_{inc}^{\pm\vec{K}}(\vec{r}_0)$ are $2(l_{max}+1)\times 1$ column vectors given by:

$$\widehat{\widehat{\mathbf{T}}}\widehat{\psi}_{\pm\vec{K}}(\vec{r}_0) = \begin{pmatrix} \psi_{\pm\vec{K}}(\vec{r}_0) \\ \widehat{T}_{1,\pm\vec{K}}\psi_{\pm\vec{K}}(\vec{r}_0) \\ \widehat{T}_{2,\pm\vec{K}}\psi_{\pm\vec{K}}(\vec{r}_0) \\ \vdots \\ \widehat{T}_{l_{max},\pm\vec{K}}\psi_{\pm\vec{K}}(\vec{r}_0) \end{pmatrix}_{\pm\vec{K}}, \widehat{\widehat{\mathbf{T}}}\widehat{\phi}_{inc}^{\pm\vec{K}}(\vec{r}_0) = \sqrt{\frac{k_1}{2v_F k_{X1}}}\begin{pmatrix} 1 \\ \pm e^{i\theta_{\vec{k}_1}} \\ e^{-i\theta_k} \\ \pm e^{2i\theta_{\vec{k}_1}} \\ e^{-2i\theta_{\vec{k}_1}} \\ \pm e^{3i\theta_{\vec{k}_1}} \\ \vdots \\ e^{-l_{max}i\theta_{\vec{k}_1}} \\ \pm e^{(l_{max}+1)i\theta_{\vec{k}_1}} \end{pmatrix}_{\pm\vec{K}} \tag{23}$$

and $\widehat{\widehat{\mathbf{TG}}}$ is a $2(l_{max}+1)\times 2(l_{max}+1)$ matrix given by:

$$\widehat{\widehat{\mathbf{TG}}} = \begin{pmatrix} s_0\tilde{G}_{0,0} & s_1\tilde{G}_{0,1} & \dots & s_{l_{max}}\tilde{G}_{0,l_{max}} \\ s_0\tilde{G}_{1,0} & s_1\tilde{G}_{1,1} & \dots & s_{l_{max}}\tilde{G}_{1,l_{max}} \\ \vdots & \vdots & \ddots & \vdots \\ s_0\tilde{G}_{l_{max},0} & s_1\tilde{G}_{l_{max},1} & \dots & s_{l_{max}}\tilde{G}_{l_{max},l_{max}} \end{pmatrix}_{\pm\vec{K}} \tag{24}$$

with

$$\begin{aligned}\tilde{G}_{l',l} &= i^{l+l'}\sum_{n\neq 0}e^{ik_{Y1}nd}\begin{pmatrix} (-1)^{l'}H^{(1)}_{l-l'}(k_1|n|d)e^{i(l-l')\theta_{0n}} & \pm iH^{(1)}_{l+l'+1}(k_1|n|d)e^{-i(l+l'+1)\theta_{0n}} \\ \pm iH^{(1)}_{l+l'+1}(k_1|n|d)e^{i(l+l'+1)\theta_{0n}} & (-1)^{l}H^{(1)}_{l'-l}(k_1|n|d)e^{i(l'-l)\theta_{0n}} \end{pmatrix}_{\pm\vec{K}} \\ &= \begin{pmatrix} S_{l-l'}(k_1d,k_{Y1}d) & \pm(-1)^{l+l'+1}S_{l+l'+1}(k_1d,k_{Y1}d) \\ \pm S_{l+l'+1}(k_1d,k_{Y1}d) & (-1)^{l-l'}S_{l-l'}(k_1d,k_{Y1}d) \end{pmatrix}_{\pm\vec{K}}\end{aligned} \tag{25}$$

where $e^{\pm i\theta_{0n}} = \mp i\frac{n}{|n|}$ and

$$S_l(k_1d,k_{Y1}d) = \sum_{n=1}^{\infty}H^{(1)}_l(k_1nd)\left(e^{ik_{Y1}nd} + (-1)^l e^{-ik_{Y1}nd}\right) \tag{26}$$

The lattice sum, $S_l(k_1 d, k_{Y1} d)$ in Eq. (26), can be efficiently calculated using a small modification to a previously published method[3] as:

$$S_l(k_1 d, k_{Y1} d) = \frac{\sqrt{2}}{\pi} e^{-i\left(\frac{\pi}{4} - k_{Y1} d\right)} \int_0^a \mathrm{d}t \frac{\left[\left(t(1-i) + i\sqrt{1+2it^2}\right)^l + \left(t(i-1) + i\sqrt{1+2it^2}\right)^l\right] e^{ik_1 d\sqrt{1+2it^2}}}{\sqrt{1+2it^2}\left(1 - e^{i\left(k_1 d\sqrt{1+2it^2} + k_{Y1} d\right)}\right)}$$
$$+ (-1)^l \frac{\sqrt{2}}{\pi} e^{-i\left(\frac{\pi}{4} + k_{Y1} d\right)} \int_0^a \mathrm{d}t \frac{\left[\left(t(1-i) + i\sqrt{1+2it^2}\right)^l + \left(t(i-1) + i\sqrt{1+2it^2}\right)^l\right] e^{ik_1 d\sqrt{1+2it^2}}}{\sqrt{1+2it^2}\left(1 - e^{i\left(k_1 d\sqrt{1+2it^2} - k_{Y1} d\right)}\right)} \tag{27}$$

In this work, the expression for $S_l(k_1 d, k_{Y1} d)$ in Eq. (27) was numerically integrated using MATLAB[4], where the upper limit of the integrals in Eq. (27) was chosen to be $a = 4000$.

Additionally, the individual sums that comprise $\widehat{G}_{l,\pm K}(\vec{r}, nd\widehat{y}, E)$ in Eq. (20) can be calculated[5] using the following plane wave expansion that is valid for $x \neq 0$:

$$\sum_{n=-\infty}^{\infty} \widehat{L}_{\pm}^{|l|}[H_0^{(1)}(k_1 \rho_n)] e^{ik_{Y1} nd} = \frac{2}{d} \sum_{n=-\infty}^{\infty} \frac{e^{i(k_{Y1}^{(n)} y + k_{X1}^{(n)}|x|)}}{k_{X1}^{(n)}} \left(\frac{k_{X1}^{(n)}\mathrm{sign}(x) \pm ik_{Y1}^{(n)}}{k_1}\right)^{|l|}$$
$$= \frac{2}{d} \sum_{n=-\infty}^{\infty} \frac{e^{i(k_{Y1}^{(n)} y + k_{X1}^{(n)}|x|)}}{k_{X1}^{(n)}} \left(\mathrm{sign}(x) e^{\pm i\mathrm{sign}(x)\theta_{\vec{k}_1^{(n)}}}\right)^{|l|} \tag{28}$$

where $k_{Y1}^{(n)} = k_{Y1} + \frac{2\pi n}{d}$, and $k_{X1}^{(n)} = \sqrt{k_1^2 - \left(k_{Y1}^{(n)}\right)^2}$ and $e^{\pm i\theta_{\vec{k}_1^{(n)}}} = \frac{k_{X1}^{(n)} \pm ik_{Y1}^{(n)}}{k_1}$ for $k_1 \geq k_{Y1}^{(n)}$, and $k_{X1}^{(n)} = i\sqrt{\left(k_{Y1}^{(n)}\right)^2 - k_1^2}$ and $e^{\pm i\theta_{\vec{k}_1^{(n)}}} = i\frac{k_{X1}^{(n)} \pm k_{Y1}^{(n)}}{k_1}$ for $k_1 \leq k_{Y1}^{(n)}$. Note that $k_{X1}^{(n)}$ will be real for $n \in \mathcal{N} = [\mathcal{N}_{\min}, \mathcal{N}_{\max}]$ where $\mathcal{N}_{\min} = \left\{\frac{-(k_1 + k_{Y1})d}{2\pi}\right\}_+$ and $\mathcal{N}_{\max} = \left\{\frac{(k_1 - k_{Y1})d}{2\pi}\right\}_-$, where $\{z\}_+$ corresponds to the smallest integer greater than z, and $\{z\}_-$ corresponds to the largest integer less than z.

In this case, $\psi_{\pm\vec{K}}(\vec{r})$ in Eq. (20) can be written as [for $x \neq 0$]:

$$\psi_{\pm\vec{K}}(\vec{r}) = \phi_{inc}^{\pm\vec{K}}(\vec{r})$$
$$+ \sum_{l=0}^{l_{max}} \sum_{n=-\infty}^{\infty} \frac{2s_l}{d} \frac{e^{i(k_{Y1}^{(n)} y + k_{X1}^{(n)}|x|)}}{k_{X1}^{(n)}} (\mathrm{sign}(x))^l \begin{pmatrix} e^{i\mathrm{sign}(x) l\theta_{\vec{k}_1^{(n)}}} & \pm\mathrm{sign}(x) e^{-i\mathrm{sign}(x)(l+1)\theta_{\vec{k}_1^{(n)}}} \\ \pm\mathrm{sign}(x) e^{i\mathrm{sign}(x)(l+1)\theta_{\vec{k}_1^{(n)}}} & e^{-i\mathrm{sign}(x) l\theta_{\vec{k}_1^{(n)}}} \end{pmatrix}_{\pm\vec{K}} \widehat{T}_{l,\pm\vec{K}} \psi_{\pm\vec{K}}(\vec{r}_0) \tag{29}$$

In Eq. (29), $\psi_{\pm\vec{K}}(\vec{r})$ consists of a series of plane waves for $n \in \mathcal{N}$ that are either transmitted [$x > 0$] or reflected [$x < 0$] from the scattering array along with evanescent waves along the $\widehat{x}$-direction that are freely propagating along the $\widehat{y}$-direction for $n \notin \mathcal{N}$. These types of evanescent waves have been predicted to exist in graphene for one-dimensional quantum wells[6] for certain values of

k_{Y1}, quantum well potential and width. For the scattering array, however, it is the periodicity of the one-dimensional array of scatterers that generates the evanescent waves in $\psi_{\pm\vec{K}}(\vec{r})$.

Finally, it should be noted that calculating $\psi_{\pm\vec{K}}(\vec{r})$ using a plane wave expansion in Eq. (29) is computationally efficient for $|x| \geq d$ since the number of plane and evanescent waves that significantly contribute to $\psi_{\pm\vec{K}}(\vec{r})$ is on the order of $O\left[\mathscr{N}_+ - \mathscr{N}_-\right]$. However, the number of evanescent waves that contribute significantly to $\psi_{\pm\vec{K}}(\vec{r})$ increases dramatically as $|x| \to 0$, which renders the plane wave expansion in Eq. (29) as an inefficient method to calculate $\psi_{\pm\vec{K}}(\vec{r})$. In this case, the sum of hankel functions in Eq. (28) should be explicitly evaluated. Note also that although the expression for $\psi_{\pm\vec{K}}(x\widehat{x}+y\widehat{y})$ in Eq. (29) is valid only for $x \neq 0$, the wave function is continuous at $x=0$ since $\lim_{x\to 0-} \psi_{\pm\vec{K}}(x\widehat{x}+y\widehat{y}) = \lim_{x\to 0+} \psi_{\pm\vec{K}}(x\widehat{x}+y\widehat{y})$ for $y \neq nd$, which can be seen using the hankel sum expressions for $S_l(k_1 d, k_{Y1} d)$ in Eq. (26).

V. TRANSMISSION AND REFLECTION FROM A ONE-DIMENSIONAL ARRAY OF SCATTERERS IN GRAPHENE

From Eq. (29), the transmitted wave function $[x > 0]$ will consist of all plane waves in $\psi_{\pm\vec{K}}(\vec{r}$ in Eq. (29) that are scattered along the Bragg directions, $\vec{k}_1^{(n)} = k_{X1}^{(n)}\widehat{x} + k_{Y1}^{(n)}\widehat{y}$ for $n \in \mathscr{N}$, $\psi_T^{\pm\vec{K}}(\vec{r})$, which can be written as:

$$\psi_T^{\pm\vec{K}}(\vec{r}) = \sum_{n\in\mathscr{N}} T_n e^{i(k_{Y1}^{(n)} y + k_{X1}^{(n)} x)} \sqrt{\frac{k_1}{2v_F k_{X1}^{(n)}}} \begin{pmatrix} 1 \\ \pm e^{i\theta_{\vec{k}_1^{(n)}}} \end{pmatrix}_{\pm\vec{K}} \tag{30}$$

where the sum in Eq. (30) is over all open channels, $n \in \mathscr{N}$, with the transmission coefficient for the nth open channel given by:

$$\begin{aligned} T_n &= \delta_{n0} + \sum_{l=0}^{l_{max}} \frac{s_l}{d} \sqrt{\frac{2v_F}{k_1 k_{X1}^{(n)}}} \left[\begin{pmatrix} 1 \\ \pm e^{i\theta_{\vec{k}_1^{(n)}}} \end{pmatrix}_{\pm\vec{K}} \right]^{\dagger} \begin{pmatrix} e^{il\theta_{\vec{k}_1^{(n)}}} & \pm e^{-i(l+1)\theta_{\vec{k}_1^{(n)}}} \\ \pm e^{i(l+1)\theta_{\vec{k}_1^{(n)}}} & e^{-il\theta_{\vec{k}_1^{(n)}}} \end{pmatrix}_{\pm\vec{K}} \widehat{T}_{l,\pm\vec{K}} \psi_{\pm\vec{K}}(\vec{r}_0) \\ &= \delta_{n0} + \sum_{l=0}^{l_{max}} \frac{2s_l}{d} \sqrt{\frac{2v_F}{k_1 k_{X1}^{(n)}}} \left[\begin{pmatrix} e^{il\theta_{\vec{k}_1^{(n)}}} \\ \pm e^{-i(l+1)\theta_{\vec{k}_1^{(n)}}} \end{pmatrix}_{\pm\vec{K}} \right]^{\mathrm{T}} \widehat{T}_{l,\pm\vec{K}} \psi_{\pm\vec{K}}(\vec{r}_0) \end{aligned} \tag{31}$$

where δ_{ij} is the Kronecker delta ($\delta_{ij} = 0$ for $i \neq j$ and $\delta_{ij} = 1$ for $i = j$). The total transmission probability is given by $T_{tot} = \sum_{n\in\mathscr{N}} |T_n|^2$.

Likewise, the reflected wave function ($x < 0$), $\psi_R^{\pm\vec{K}}(\vec{r})$, is given by:

$$\psi_R^{\pm\vec{K}}(\vec{r}) = \sum_{n\in\mathscr{N}} R_n e^{i(k_{Y1}^{(n)}y - k_{X1}^{(n)}x)} \sqrt{\frac{k_1}{2\nu_F k_{X1}^{(n)}}} \begin{pmatrix} 1 \\ \mp e^{-i\theta_{\vec{k}_1^{(n)}}} \end{pmatrix}_{\pm\vec{K}} \tag{32}$$

where

$$R_n = \sum_{l=0}^{l_{max}} \frac{(-1)^l s_l}{d} \sqrt{\frac{2\nu_F}{k_1 k_{X1}^{(n)}}} \left[\begin{pmatrix} 1 \\ \mp e^{i\theta_{\vec{k}_1^{(n)}}} \end{pmatrix}_{\pm\vec{K}} \right]^\dagger \begin{pmatrix} e^{-il\theta_{\vec{k}_1^{(n)}}} & \mp e^{i(l+1)\theta_{\vec{k}_1^{(n)}}} \\ \mp e^{-i(l+1)\theta_{\vec{k}_1^{(n)}}} & e^{il\theta_{\vec{k}_1^{(n)}}} \end{pmatrix}_{\pm\vec{K}} \widehat{T}_{l,\pm\vec{K}} \Psi_{\pm\vec{K}}(\vec{r}_0)$$

$$= \sum_{l=0}^{l_{max}} \frac{(-1)^l 2 s_l}{d} \sqrt{\frac{2\nu_F}{k_1 k_{X1}^{(n)}}} \left[\begin{pmatrix} e^{-il\theta_{\vec{k}_1^{(n)}}} \\ \mp e^{i(l+1)\theta_{\vec{k}_1^{(n)}}} \end{pmatrix}_{\pm\vec{K}} \right]^{\mathrm{T}} \widehat{T}_{l,\pm\vec{K}} \Psi_{\pm\vec{K}}(\vec{r}_0) \tag{33}$$

The transmission and reflection coefficients satisfy the unitarity condition, $\sum_{n\in\mathscr{N}} |R_n|^2 + |T_n|^2 = 1$.

VI. SCATTERED WAVE FUNCTION FROM AN INFINITE ONE-DIMENSIONAL ARRAY OF SCATTERERS IN A TWO-DIMENSIONAL ELECTRON GAS (2DEG)

In the following, we generalize previous work[7] on scattering of plane waves from a one-dimensional periodic grating in a 2DEG to include higher partial waves [$l > 0$] for comparison with the results derived for graphene. Consider an incident wave with effective mass m and energy $E = \sqrt{\frac{\hbar^2 k_1^2}{2m}}$ and normalized to unit flux along the $\widehat{x}-$direction, $\phi_{inc}^{\mathrm{ac}}(\vec{r}) = \sqrt{\frac{m}{\hbar k_{X1}}} e^{-i\vec{k}_1\cdot\vec{r}}$, where $\vec{k}_1 = k_{X1}\widehat{x} + k_{Y1}\widehat{y} = k_1 \cos(\theta_{\vec{k}_1})\widehat{x} + k_1 \sin(\theta_{\vec{k}_1})\widehat{y}$ is the wave vector.

In this case, the total wave function, $\psi^{\mathrm{ac}}(\vec{r})$, is given by [for $x \neq 0$]:

$$\psi^{\mathrm{ac}}(\vec{r}) = \phi_{inc}^{\mathrm{ac}}(\vec{r}) + \sum_{n=-\infty}^{\infty} e^{ik_{Y1}nd} \sum_{l=-l_{max}}^{l_{max}} s_{|l|}^{ac} \widehat{L}_{\mathrm{sign}(l)}^{|l|} \left[H_0^{(1)}(k_1\rho_n) \right] \widehat{L}_{-\mathrm{sign}(l)}^{|l|} [\psi^{\mathrm{ac}}(\vec{r}_0)]$$

$$= \phi_{inc}^{\mathrm{ac}}(\vec{r}) + \sum_{l=-l_{max}}^{l_{max}} \sum_{n=-\infty}^{\infty} \frac{2 s_{|l|}^{ac}}{d} \frac{e^{i(k_{Y1}^{(n)}y + k_{X1}^{(n)}|x|)}}{k_{X1}^{(n)}} (\mathrm{sign}(x))^l e^{i\mathrm{sign}(x) l \theta_{\vec{k}_1^{(n)}}} \widehat{L}_{-\mathrm{sign}(l)}^{|l|} [\psi^{\mathrm{ac}}(\vec{r}_0)] \tag{34}$$

where again $k_{Y1}^{(n)} = k_{Y1} + \frac{2\pi n}{d}$, and $k_{X1}^{(n)} = \sqrt{k_1^2 - \left(k_{Y1}^{(n)}\right)^2}$ and $e^{\pm i\theta_{\vec{k}_1^{(n)}}} = \frac{k_{X1}^{(n)} \pm i k_{Y1}^{(n)}}{k_1}$ for $k_1 \geq k_{Y1}^{(n)}$, and $k_{X1}^{(n)} = i\sqrt{\left(k_{Y1}^{(n)}\right)^2 - k_1^2}$ and $e^{\pm i\theta_{\vec{k}_1^{(n)}}} = i\frac{k_{X1}^{(n)} \pm k_{Y1}^{(n)}}{k_1}$ for $k_1 \leq k_{Y1}^{(n)}$. For $n \in \mathscr{N} = [\mathscr{N}_{\min}, \mathscr{N}_{\max}]$ where $\mathscr{N}_{\min} = \left\{ \frac{-(k_1 + k_{Y1})d}{2\pi} \right\}_+$ and $\mathscr{N}_{\max} = \left\{ \frac{(k_1 - k_{Y1})d}{2\pi} \right\}_-$, $k_{X1}^{(n)}$ is real. Furthermore, the various $\widehat{L}_{\pm}^{|l|}[\psi^{\mathrm{ac}}(\vec{r}_0)]$ are determined from solving the following equation:

$$\left(\widehat{\widehat{\mathbf{1}}} - \widehat{\widehat{\mathbf{TG}}}^{\mathrm{ac}} \right) \widehat{\widehat{\mathbf{T}}} \widehat{\psi}^{\mathrm{ac}}(\vec{r}_0) = \widehat{\widehat{\mathbf{T}}} \widehat{\phi}_{inc}^{\mathrm{ac}}(\vec{r}_0) \tag{35}$$

where $\widehat{\widehat{\mathbf{1}}}$ is a $(2l_{max}+1)\times(2l_{max}+1)$ identity matrix, $\widehat{\widehat{\mathbf{TG}}}^{\text{ac}}$ is a $(2l_{max}+1)\times(2l_{max}+1)$ matrix with the (m,n) element given by $\left(\widehat{\widehat{\mathbf{TG}}}^{\text{ac}}\right)_{m,n} = s^{ac}_{|l_{max}+1-n|} S_{m-n}(k_1 d, k_{Y1} d)$. In this case, $\widehat{\widehat{\mathbf{TG}}}^{\text{ac}}$ can be explicitly written as:

$$\widehat{\widehat{\mathbf{TG}}}^{\text{ac}} = \begin{pmatrix} s^{ac}_{l_{max}} S_0(k_1 d,k_{Y1}d) & s^{ac}_{l_{max}-1} S_{-1}(k_1 d,k_{Y1}d) & \dots & s^{ac}_{0} S_{-l_{max}}(k_1 d,k_{Y1}d) & \dots & s^{ac}_{l_{max}-1} S_{-2l_{max}+1}(k_1 d,k_{Y1}d) & s^{ac}_{l_{max}} S_{-2l_{max}}(k_1 d,k_{Y1}d) \\ s^{ac}_{l_{max}} S_1(k_1 d,k_{Y1}d) & s^{ac}_{l_{max}-1} S_{0}(k_1 d,k_{Y1}d) & \dots & s^{ac}_{0} S_{-l_{max}+1}(k_1 d,k_{Y1}d) & \dots & s^{ac}_{l_{max}-1} S_{-2l_{max}+2}(k_1 d,k_{Y1}d) & s^{ac}_{l_{max}} S_{-2l_{max}+1}(k_1 d,k_{Y1}d) \\ \vdots & \vdots & \ddots & \vdots & \ddots & \vdots & \vdots \\ s^{ac}_{l_{max}} S_{2l_{max}-1}(k_1 d,k_{Y1}d) & s^{ac}_{l_{max}-1} S_{2l_{max}-2}(k_1 d,k_{Y1}d) & \dots & s^{ac}_{0} S_{l_{max}-1}(k_1 d,k_{Y1}d) & \dots & s^{ac}_{l_{max}-1} S_{0}(k_1 d,k_{Y1}d) & s^{ac}_{l_{max}} S_{-1}(k_1 d,k_{Y1}d) \\ s^{ac}_{l_{max}} S_{2l_{max}}(k_1 d,k_{Y1}d) & s^{ac}_{l_{max}-1} S_{2l_{max}-1}(k_1 d,k_{Y1}d) & \dots & s^{ac}_{0} S_{l_{max}}(k_1 d,k_{Y1}d) & \dots & s^{ac}_{l_{max}-1} S_{1}(k_1 d,k_{Y1}d) & s^{ac}_{l_{max}} S_{0}(k_1 d,k_{Y1}d) \end{pmatrix} \tag{36}$$

where $S_l(k_1 d, k_{Y1} d)$ can be evaluated using Eq. (27), and:

$$\widehat{\widehat{\mathbf{T}}}\widehat{\psi}^{\text{ac}}(\vec{r}_0) = \begin{pmatrix} \widehat{L}^{l_{max}}\left[\psi^{\text{ac}}(\vec{r}_0)\right] \\ \widehat{L}_{-}^{l_{max}-1}\left[\psi^{\text{ac}}(\vec{r}_0)\right] \\ \vdots \\ \psi^{\text{ac}}(\vec{r}_0) \\ \vdots \\ \widehat{L}_{+}^{l_{max}-1}\left[\psi^{\text{ac}}(\vec{r}_0)\right] \\ \widehat{L}_{+}^{l_{max}}\left[\psi^{\text{ac}}(\vec{r}_0)\right] \end{pmatrix}, \widehat{\widehat{\mathbf{T}}}\widehat{\phi}^{\text{ac}}_{inc}(\vec{r}_0) = e^{i\vec{k}_1\cdot\vec{r}_0}\sqrt{\frac{m}{\hbar k_{X1}}} \begin{pmatrix} e^{-il_{max}\theta_{\vec{k}_1}} \\ e^{-i(l_{max}-1)\theta_{\vec{k}_1}} \\ \vdots \\ 1 \\ \vdots \\ e^{i(l_{max}-1)\theta_{\vec{k}_1}} \\ e^{il_{max}\theta_{\vec{k}_1}} \end{pmatrix} \tag{37}$$

For a scattering potential given by $\widehat{V}_{ac}(\vec{r},E) = \sum_{n=-\infty}^{\infty} V_{ac}(E)\Theta(|\vec{r}-\vec{r}_n|)$ where $\Theta(z)=1$ for $z\le r_s$ and $\Theta(z)=0$ for $z>r_s$, the scattering amplitudes are given by

$$s^{ac}_l = \frac{J_l(k_1 r_s)J_{l+1}(k_2 r_s) - J_l(k_2 r_s)J_{l+1}(k_1 r_s)}{k_1 H^{(1)}_{l+1}(k_1 r_s)J_l(k_2 r_s) - k_2 J_{l+1}(k_2 r_s)H^{(1)}_l(k_1 r_s)} \tag{38}$$

where $k_1 = \sqrt{\frac{2mE}{\hbar^2}}$, and $k_2 = \sqrt{\frac{2m(E-V_{ac}(E))}{\hbar^2}}$ for $E \ge V_{ac}(E)$ or $k_2 = i\sqrt{\frac{2m|E-V_{ac}(E)|}{\hbar^2}}$ for $E < V_{ac}(E)$. Note that $s^{ac}_l = s^{ac}_{-l}$ in Eq. (38). For comparing the calculations in a 2DEG to the calculations in graphene, the magnitudes of k_1 and k_2 for the 2DEG were chosen to be the same as in the graphene [details are given in the caption of Figure 3 in the main manuscript].

In this case, the transmitted and reflected wave functions, $\psi^{\text{ac}}_T(\vec{r})$ for $x>0$ and $\psi^{\text{ac}}_R(\vec{r})$ for $x<0$, are determined from Eq. (34):

$$\psi^{\text{ac}}_T(\vec{r}) = \sum_{n\in\mathcal{N}} T^{\text{ac}}_n e^{i(k^{(n)}_{Y1}y + k^{(n)}_{X1}x)}\sqrt{\frac{m}{\hbar k^{(n)}_{X1}}}$$
$$\psi^{\text{ac}}_R(\vec{r}) = \sum_{n\in\mathcal{N}} R^{\text{ac}}_n e^{i(k^{(n)}_{Y1}y - k^{(n)}_{X1}x)}\sqrt{\frac{m}{\hbar k^{(n)}_{X1}}} \tag{39}$$

where the reflection and transmission coefficients satisfy the unitarity condition, $\sum_{n\in\mathscr{N}} |T_n^{\rm ac}|^2 + |R_n^{\rm ac}|^2 = 1$, and are given by:

$$T_n^{\rm ac} = \delta_{n0} + \sum_{l=-l_{max}}^{l_{max}} \frac{2s_{|l|}^{ac}}{d}\sqrt{\frac{m}{\hbar k_{X1}^{(n)}}} e^{il\theta_{\vec{k}_1^{(n)}}} \widehat{L}_{-\mathrm{sign}(l)}^{|l|}\left[\psi^{\rm ac}(\vec{r}_0)\right]$$
$$R_n^{\rm ac} = \sum_{l=-l_{max}}^{l_{max}} \frac{2s_{|l|}^{ac}(-1)^l}{d}\sqrt{\frac{m}{\hbar k_{X1}^{(n)}}} e^{-il\theta_{\vec{k}_1^{(n)}}} \widehat{L}_{-\mathrm{sign}(l)}^{|l|}\left[\psi^{\rm ac}(\vec{r}_0)\right] \tag{40}$$

Like in graphene, l_{max} was chosen to take into account 99.9% of the total scattering amplitude for an individual scatterer in the 2DEG, i.e., $\sum_{l=-l_{max}}^{l_{max}} \left|s_l^{ac}\right|^2 \approx 0.999 \sum_{l=-\infty}^{\infty} \left|s_l^{ac}\right|^2$.

ACKNOWLEDGMENTS

This work was supported by the National Science Foundation under CHE - 1056846 and from funds from the University of Miami.

---

[1] Vaishnav, J. Y., Anderson, J. Q. & Walls, J. D. Intravalley multiple scattering of quasiparticles in graphene. *Phys. Rev. B* **83**, 165437 (2011).

[2] Katsnelson, M. I. & Novoselov, K. S. Graphene: New bridge between condensed matter physics and quantum electrodynamics. *Sol. State Comm.* **143**, 3–13 (2007).

[3] Yasumoto, K. & Yoshitomi, K. Efficient calculation of lattice sums for free-space periodic green's function. *IEEE Trans. Antennas Propagat.* **47**, 1050–1055 (1999).

[4] Mathworks. Matlab. `http://www.mathworks.com`.

[5] Nicorovici, N. A., McPhedran, R. C. & Petit, R. Efficient calculation of the green's function for electromagnetic scattering by gratings. *Phys. Rev. E* **49**, 4563–4577 (1994).

[6] Pereira, J. M., Mlinar, V., Peeters, F. M. & Vasilopoulos, P. Confined states and direction-dependent transmission in graphene quantum wells. *Phys. Rev. B* **74**, 045424 (2006).

[7] Vaishnav, J. Y., Walls, J. D., Apratim, M. & Heller, E. J. Matter-wave scattering and guiding by atomic arrays. *Phys. Rev. A* **76**, 013620 (2007).
